# Microbiome and resistome of the European bison (*Bison bonasus*)

**DOI:** 10.1038/s41598-026-64319-9

**Published:** 2026-08-06

**Authors:** Nele Lechleiter, Judith Wedemeyer, Jessica Junker, Magdalena Wilczek, Daniel Klich, Wanda Olech, Krzysztof Anusz, Timo Homeier-Bachmann, Anna Didkowska

**Affiliations:** 1 Institute of Epidemiology, Friedrich-Loeffler-Institut, Island of Riems, Südufer 10, 17493 Greifswald, Germany; 2 Department of Food Hygiene and Public Health Protection, Institute of Veterinary Medicine, Warsaw University of Life Sciences (WULS-SGGW), Nowoursynowska 159 St, 02-787 Warsaw, Poland; 3 Department of Animal Genetics and Conservation, Institute of Animal Sciences, Warsaw University of Life Sciences (WULS-SGGW), Warsaw, Poland

**Keywords:** European bison, Microbiome, Rresistome, Genetics, Microbiology

## Abstract

After facing extinction in the early 20th century, populations of the two remnant genetic lines of European bison are now under continuous health monitoring. Faecal samples were taken from five Polish and one German herd of European bison over the course of several years. Through metagenomic sequencing, the bacterial and archaeal microbiome as well as the resistome of these samples could be characterized. Significant differences were mainly found between the bacterial microbiome of samples taken from droppings as opposed to rectal samples. Apart from this, the microbiome and resistome had low differentiation, showing no significant influence of individual factors or location. Oscillospiraceae, Lachnospiraceae and Bacteroidaceae were the dominant bacterial families, the archaeome was mostly made up by Methanobacteriaceae. Genes from resistance classes like Aminoglycosides and Macrolide, Lincosamide and Streptogramine were present. This study characterises the microbiome and resistome of the European bison with the help of metagenomics, providing novel insights into its biology.

**Supplementary Information:**

The online version contains supplementary material available at 10.1038/s41598-026-64319-9.

## Introduction

The European bison (*Bison bonasus*), the largest terrestrial mammal in Europe, represents a flagship species for wildlife conservation. After near extinction in the early 20th century, the species has been successfully restored through intensive conservation programs^1^. At present, the European bison population is divided into two breeding lines: the lowland line, consisting of genetically pure *Bison bonasus bonasus* and originating from seven founders, and the lowland–Caucasian line, derived from crosses between *Bison bonasus bonasus* and *Bison bonasus caucasicus* and esTablelished from twelve founder individuals^2–4^. The European bison population in Europe currently numbers over 12,200 individuals, with approximately 3,060 occurring in Poland^5^. Despite recovery, European bison populations remain highly susceptible to environmental stressors^6^ and pathogens^7,8^. This could be an effect of increasing population density in recent years^1^, and results in the necessity of continuous health monitoring as a key component of conservation strategies^9^. In recent years, increasing attention has been paid to the role of the microbiome in host health and disease susceptibility^10,11^. The animal microbiome plays a crucial role in immune system development, meTableolic processes, and protection against pathogens^12^. In ruminants, the gut microbiome is particularly important due to its involvement in digestion and nutrient utilization^13^. However, knowledge of the microbiome composition in European bison remains limited. A study on European bison in a national park in the Netherlands described the dominant phyla and classes in the faecal microbiome and outlined compositional differences to sympatric herbivores^14^. Here, Firmicutes (/Bacillota), Bacteroidota, Proteobacteria (/Pseudomonadota) and Verrucomicrobiota are described as most abundant. The composition was found to be similar to cows and fallow deer. If this pattern is consistent across populations and not induced by the sympatry of the compared species remains open. Following the “One Health” concept there is high need for studies on anthropogenic influence on the environment, including antimicrobial resistance genes (AMRG) in wildlife^14,15^. Especially under aspects of reintroduction, detailed knowledge about AMRG abundance is required to allow for more targeted monitoring and management programs. As AMRG are often carried and disseminated through gastrointestinal microbiota, faecal analyses offer insight into their composition and abundance^15^. Recent studies have used cultivation-based techniques, focusing investigations on the AMRG solely in *Escherichia coli*^16^. Overall, a point of reference is needed as the basis of future investigations. By sampling across multiple populations, this study provides a definition of general patterns in the microbiome and resistome, while investigating possible influential factors. The existing studies provide taxonomic and functional insights using 16S rDNA sequencing, whereas shotgun metagenomics additionally enables the characterization of archaeal communities and antimicrobial resistance genes. In this context, this study aims to characterize the microbiome and resistome of the European bison, providing insights into microbial diversity and the occurrence of resistance genes.

## Materials and methods

### Sampling

Material was collected from animals originating from one German and five Polish herds (Table. 1).

**Table. 1:** Sample number of acquired samples.

	Białowieska forest (PL)	Bieszczady mountains (PL)	Borecka forest (PL)	Döberitz (DE)	Knyszyńska forest (PL)	Pszczyna (PL)
no. of samples	13	25	1	28	10	1

Faecal samples from Poland were collected from 50 European bison (26 females, 24 males). The study included European bison populations originating from four free living herds in Poland: the Bieszczady Mountains (samples collected in 2020 and 2022–2024), the Białowieska Forest (2021 and 2023–2024), the Knyszyńska Forest (2023–2025), and the Borecka Forest (2023), as well as from the one semi-free-ranging population maintained in Pszczyna (2018). The Białowieska, Borecka, and Knyszyńska Forests represent lowland populations located in north-eastern Poland, whereas the Bieszczady Mountains population inhabits a mountainous region in south-eastern Poland. The Pszczyna herd, located in southern Poland, constitutes one of the oldest European bison breeding centers in the country. All studied animals originated from non-vaccinated populations. All studied populations were subject to management practices involving seasonal supplemental feeding, particularly during winter, which is commonly applied in European bison conservation programs in Poland. No animal was culled or immobilized for the sole purpose of this study and material was collected from the rectum while performing other procedures. All procedures, including the culling of European bison, were conducted with approval from the General Directorate for Environmental Protection in Poland, in accordance with the Act of 16 April 2004 on Nature Conservation. The collection and long-term storage of biological material were authorized by a decision issued by the Regional Director for Environmental Protection in Warsaw. Faecal samples were collected by qualified veterinarians directly from the rectum of animals subjected to immobilization or culling. The samples were subsequently transported to the laboratory and stored at −20 °C until further analyses were performed.

Döberitz – Sampling took place in the Sielmann’s nature reserve Döberitz Heath on the 18^th^ of April and 30^th^ of October 2024. On a fenced area of over 1000 ha, more than 100 European bison were released from 2008 onwards, living in sympatry with German native wildlife and Przewalski’s horses. The animals are self-sufficient and do not receive vaccinations or supplementary feed. With the help of park rangers, fresh droppings of European bison were opportunistically collected across the area. Since animals were not observed before sample collection, a repeated sampling of the same individuals cannot be excluded. Furthermore, no metadata for these samples is available apart from the year of collection. Metadata for all samples can be accessed in supplementary Table 1.

### Sampling outcome

A total of 78 samples were collected (Table.1) and could be subjected to further analysis. App.1 provides an overview of the available metadata connected to the individual samples. Age groups were determined as follows: 0 = calf, 0–1 y; 1 = immature, 1–3 y; 2 = young adult, 3–10 y; 3 = mature adult, 10–20 y; 4 = aged, ≥ 20 y.

### Metagenomic sequencing and analysis

The protocol for metagenomic sequencing and analysis was adapted from Lechleiter et al^17^.. Following most of the standard protocol, the QIAamp Fast DNA Stool Mini Kit (QIAGEN, Hilden, Germany) was used to extract DNA from 0.4 g of faecal matter. Some modifications to the process were adapted from Knudsen et al^18^. Specifically, an additional extraction step with a FastPrep (MP Biomedicals, Santa Ana, CA, USA) at 30 Hz in 3 pulses of 30 s each, 95 °C for lysis and a higher volume (30 µl) of Proteinase K. A QuBit Fluorometer (Thermofisher Scientific, Waltham, MA, USA) was used to measure the concentration of the extracted DNA with the QuBit dsDNA HS Assay Kit (Thermofisher Scientific, Waltham, MA, USA). The Illumina sequencing was performed by SeqCenter (SeqCenter, Pittsburgh, PA, USA) on a NovaSeq 6000 sequencer (Illumina Inc., San Diego, CA, USA). For library preparation, the Illumina DNA Prep kit and unique dual indices were used, sequencing produced 2x151bp paired-end-reads. For the resistome analysis, the AMR++ pipeline (v3.0) was used with default parameters and the MEGARes v3.0 daTablease^17^. Bases of low quality and host DNA are removed within this pipeline. As host reference genome, the NCBI reference genome for cattle (GCA_002263795.4) was chosen, as no assembled genome for European bison was available at the time. As output, the AMR sequence hits on the level of antibiotic classes, resistance mechanisms and corresponding genes are produced. METAXA 2 (version 2.2.3)^18^was used to determine the number of bacterial 16S rDNA sequences in each sample, to normalize the abundance of resistance genes according to Li et al^19,20^. To assess the microbiome composition, the data was evaluated using the kraken2 pipeline (version 2.1.2)^21^ in standard parameters. Here, the reads are mapped to their lowest common ancestor in the NCBI RefSeq daTablease (version 221, build date 12.2023)^22^.

### Data analysis

R Studio (version 2023.6.2.561)^23^ in R (version 4.3.1)^24^ was used to perform statistical data analysis and tests, using the functions implemented in the “vegan” package (version 2.7-2)^25^. Differences were considered statistically significant at p < 0.05. The microbiome was analysed by filtering the kraken2 output by domain and taxonomic rank. Statistical tests between the groups were performed at family level. For the resistome analysis, the resistance gene abundance was determined using the AMR++ and METAXA2 output and differences between groups tested at class level. No rarefaction normalization was performed (App.2). Although sequencing depth varied among samples, all datasets yielded high read numbers. Thus, analyses were conducted using relative abundance data. This approach avoids discarding data and increases variance. Visualizations were created using “ggplot2” (version 4.0.2)^26^.

To characterize the alpha-diversity of the microbial taxa and AMR classes, Shannon-Index^19^, Richness and Pielou’s Evenness^20^ were calculated. A global Kruskal-Wallis test and Dunn’s test with Bonferroni correction was used to test for significant differences between the groups. For Kruskal–Wallis tests for location differences, epsilon^2^ values were calculated as measures of effect size. Factors for grouping the samples in all tests were: sex, age groups, year of sampling and sampling location. Groups with just one sample, like in some locations, were excluded from statistical tests. For beta-diversity, the Bray-Curtis dissimilarity was calculated between the samples and significant differences between the groups were tested via a PERMANOVA with 1000 permutations. Significant differences between the locations were visualized by creating an nMDS plot with the metaMDS function, contained in the vegan package.

Potential correlations between the occurrence of bacterial families and resistance classes were tested by calculating the Spearman rank correlation and testing for significant differences with a Holm correction. This analysis was performed using the “psych” package^21^.

Since the results of the statistical testing indicated differences in the bacterial composition between sampling locations, further analyses were performed to characterize these differences. The ratio of Bacillota to Bacteroidota was compared between locations using a Kruskal-Wallis test. At family and genus level, the relative abundance of the common taxa was tested for their difference in abundance at the locations. The cutoff for common taxa was set at 2% median relative abundance across samples. Dunn’s tests with Bonferroni correction were applied to determine significant differences.

## Results

### General

An average of 86,708,178 reads were produced per sample (App.2). In the taxonomic assignment via kraken2, some of the reads could only be assigned to domain level. Of the average of 11,716,673 (± 3,014,060.4) bacterial reads per sample, this was the case for 5.53% (± 1.18%). Archaea showed a generally lower read number of 222,576 (± 167,354.7) per sample, 0.01% (± 0.03) of which were not assigned further.

### Microbiome bacteria

**Fig. 1:**
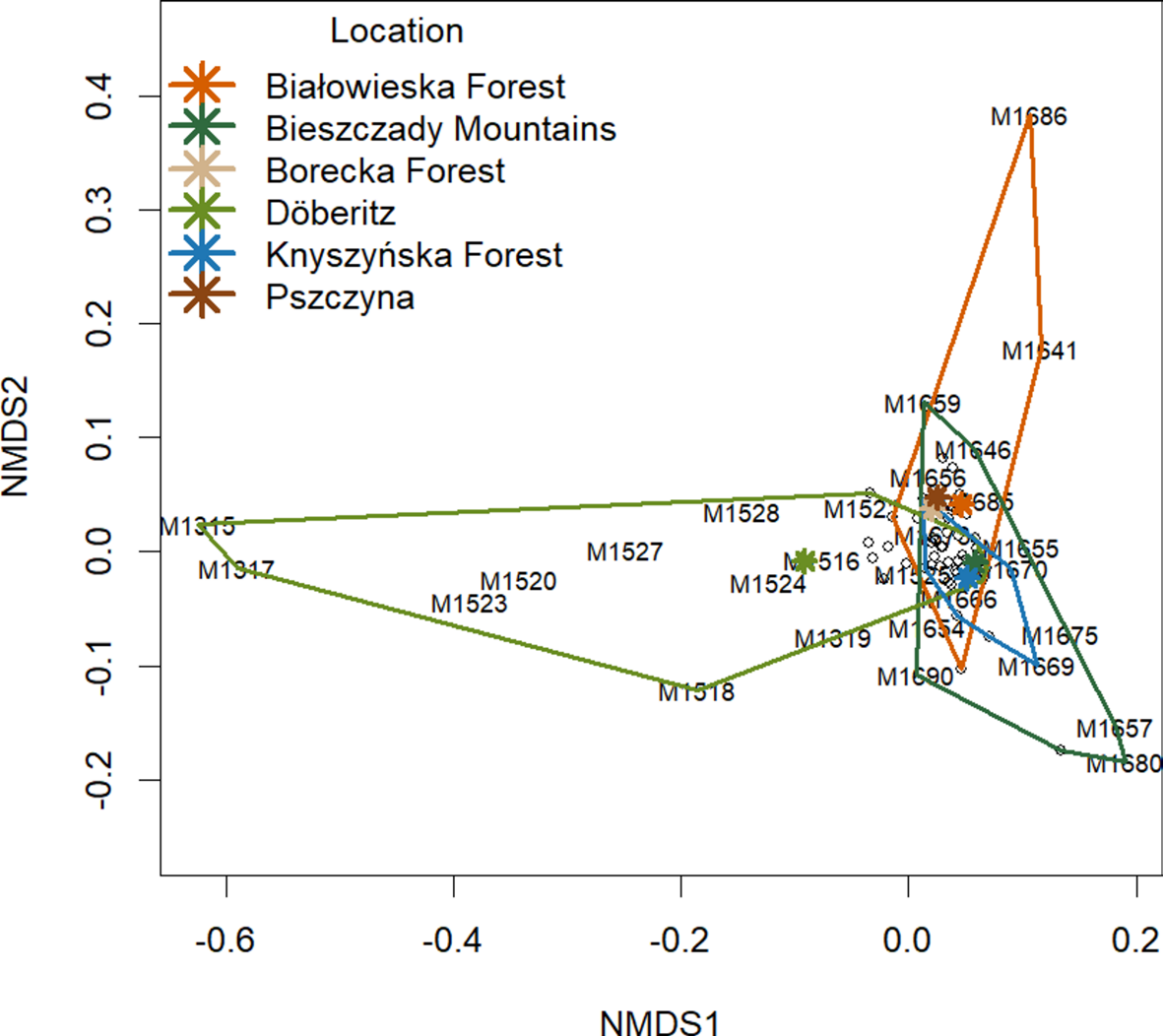
Non-metric multidimensional scaling (NMDS) based on Bray–Curtis dissimilarities calculated from bacterial relative abundance data. Each point represents an individual faecal sample, and colours indicate sampling location. To improve interpreTableility, sample labels are included, and group-wise dispersion is visualized using convex hulls. Centroids represent the mean position of each group in ordination space. Stress value of the ordination is reported in the main text/figure caption (stress = 0.044), indicating an adequate representation of the data in reduced dimensions.

Regarding the statistical tests, Döberitz had a significantly higher richness than Bieszczady Mountains (p = 0.02, ε^2^= 0.109). Because Döberitz samples were collected using a different sampling approach, they were excluded from alpha-diversity comparisons. Among the remaining locations, bacterial alpha diversity did not differ significantly. No significant effects of sex, age group or sampling year were detected either. This suggests that the observed variation was primarily associated with sampling method, rather than geographic differences between herds. In the reduced dataset, no significant differences between locations were detected for Shannon diversity (Kruskal–Wallis, p = 0.302, ε^2^ = 0.020), richness (p = 0.232, ε^2^ = 0.036), or Pielou’s evenness (p = 0.302, ε^2^ = 0.020), indicating only small effect sizes. Beta-diversity was significantly different between locations (F = 3.06, R^2^ = 0.177, p = 0.007), but not between other groups (Fig. 1, App.3).

These inferential results induced a deeper descriptive analysis of the bacterial microbiome between locations. The ratio of Bacillota to Bacteroidota did not differ significantly between locations, which may be due to the high standard deviations within the groups (Table.2).

**Table. 2:** Ratio of Bacillota to Bacteroidota and associated standard deviations per location.

	Białowieska forest	Bieszczady mountains	Borecka forest	Döberitz	Knyszyńska Forest	Pszczyna
B:B ratio	3.07	3.45	1.43	1.64	2.03	2,78
sd	1.85	1.63	NA	0.84	0.7	NA

**Fig. 2:**
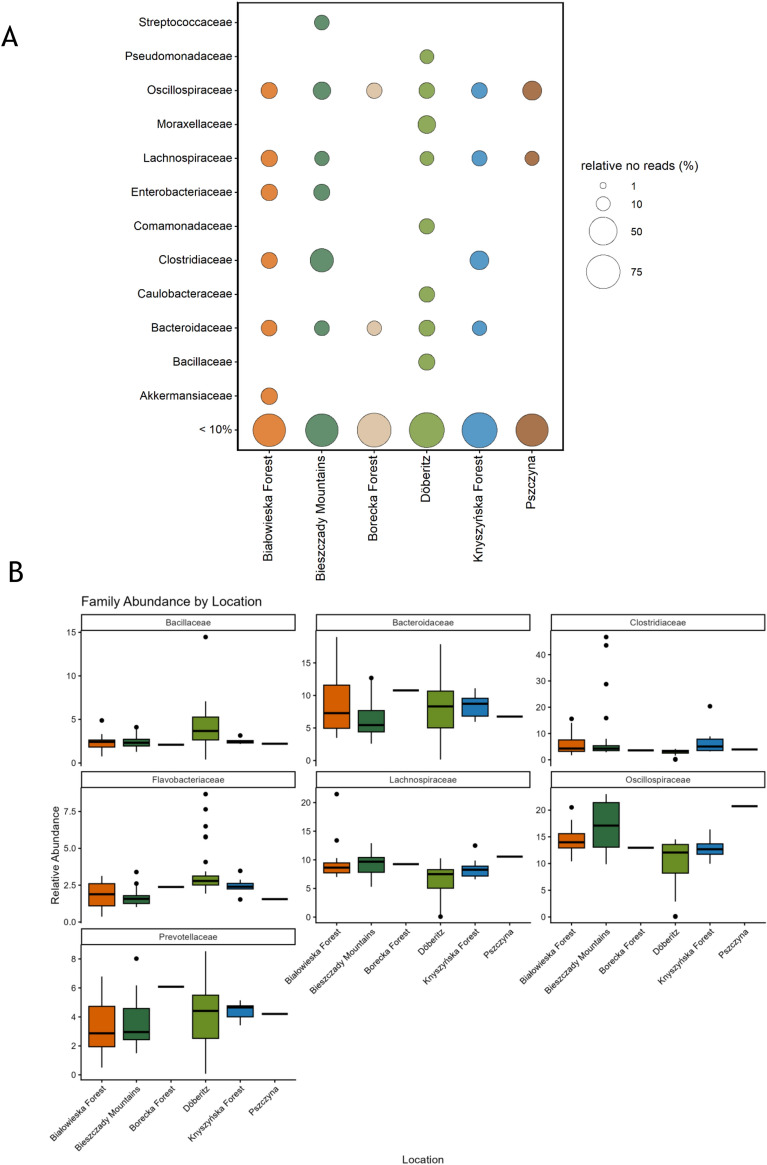
(**A**): Relative abundance of bacterial families per location. Bubble size corresponds to abundance. Families with an abundance of <10% per sample are summarized in “<10”.; (**B**): Boxplots showing the relative abundance (y-axis) of selected bacterial families by location (x-axis). Locations with just one sample (Borecka Forest, Pszczyna) and the Döberitz location (due to sampling bias) were excluded from the analysis. The boxplots represent the interquartile range (IQR), with the box showing the 25th to 75th percentiles. The line within each box marks the median relative abundance. Whiskers extend to the maximum and minimum values within 1.5 times the IQR, while points outside this range are considered outliers. Note the varying scales on the axes.

On the family level, the differences in abundance between locations were more apparent, explaining the large standard deviations in the data. There were some differences in the occurrence per location (Fig. 2A), but no statistical significances between the most common families further than Döberitz (Fig. 2B). In the samples from Döberitz, Oscillospiraceae (Döberitz-Bieszczady Mountains: p ≤ 0.001), Lachnospiraceae (Döberitz-Bieszczady Mountains: p ≤ 0.001) and Clostridiaceae (Döberitz- Bieszczady Mountains: p ≤ 0.001; Döberitz-Knyszyńska Forest: p = 0.036) were present in significantly lower numbers than in others. In contrast to that, Moraxellaceae showed higher abundances in Döberitz (Döberitz-Bieszczady Mountains/Białowieska Forest: p ≤ 0.001).

**Fig. 3:**
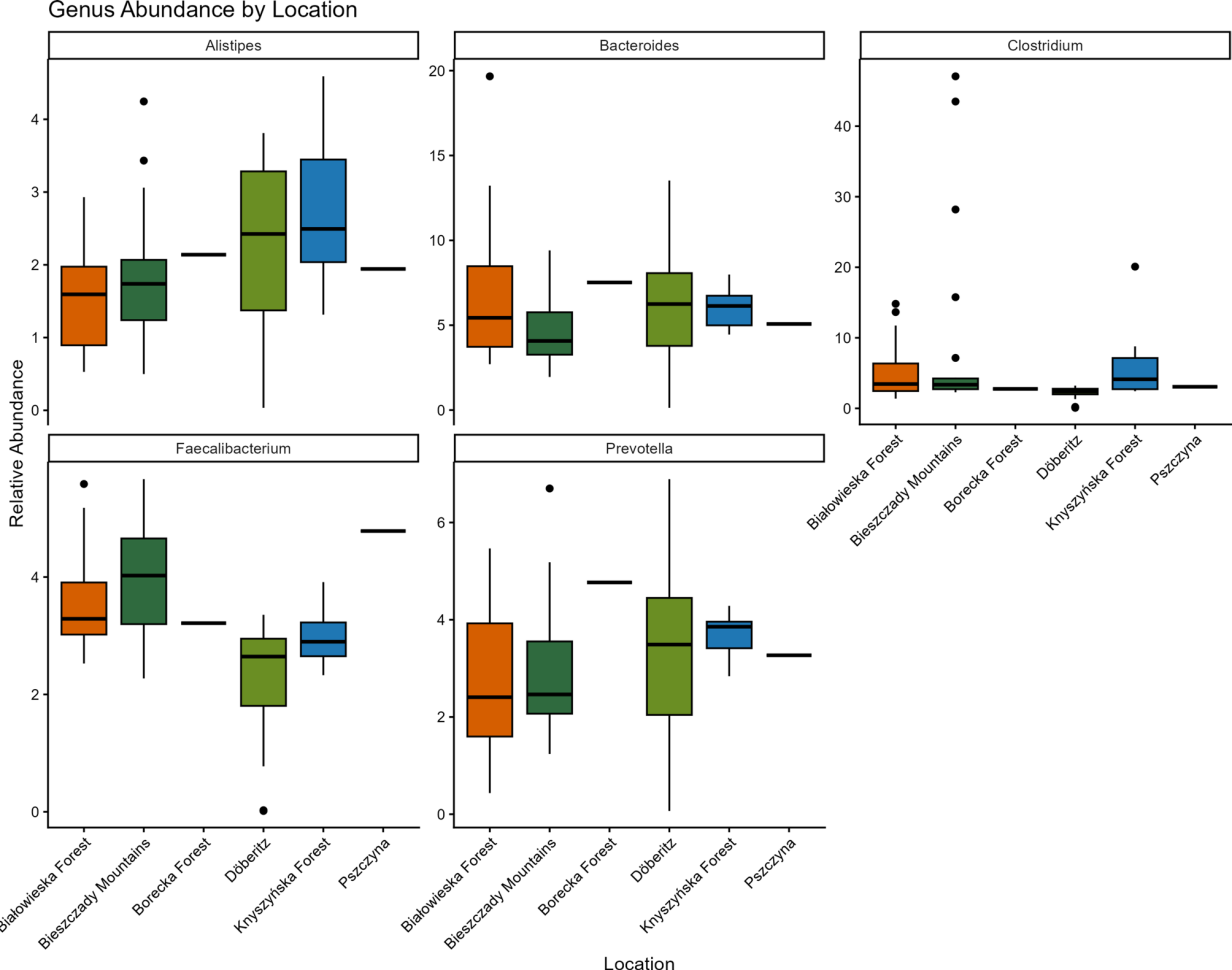
Boxplots showing the relative abundance (y-axis) of selected bacterial genera by location (x-axis). Locations with just one sample (Borecka Forest, Pszczyna) and the Döberitz location (due to sampling bias) were excluded from the analysis. The boxplots represent the interquartile range (IQR), with the box showing the 25th to 75th percentiles. The line within each box marks the median relative abundance. Whiskers extend to the maximum and minimum values within 1.5 times the IQR, while points outside this range are considered outliers.

**Fig. 4:**
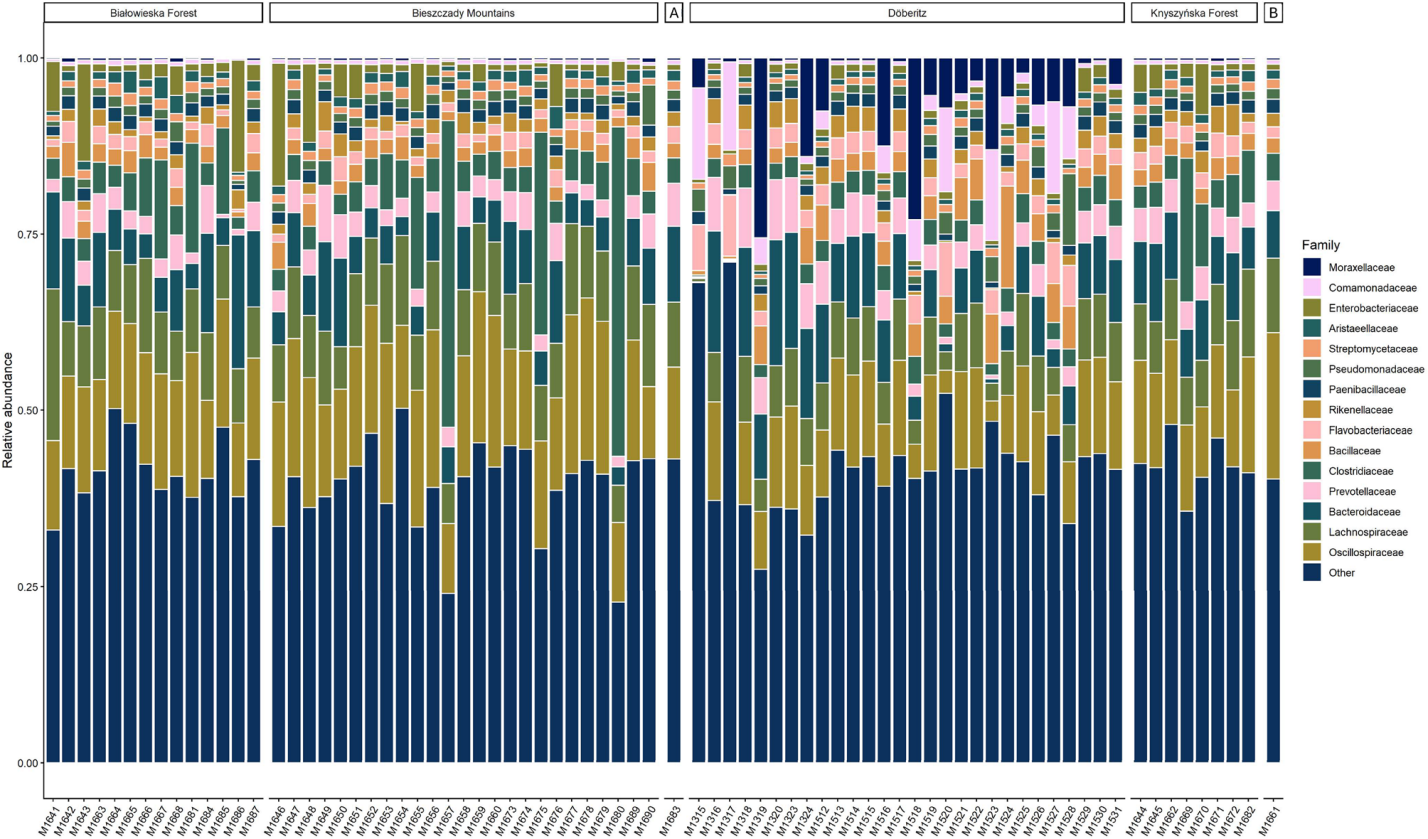
Relative abundance of the 15 most abundant bacterial families across samples, gouped by sampling location. Taxa outside the 15 most abundant families were combined into the category “Other”. Relative abundances are shown as percentages of total bacterial reads per sample. A indicates the sample from Borecka Forest, B from Pszczyna.

Similar effects were to be found when testing the most abundant genera (Fig. 3). Again, most deviating was Döberitz, with lower numbers of *Clostridium* (Döberitz-Bieszczady Mountains: p = 0.001, Döberitz-Knyszyńska Forest: p = 0.034), *Vescimonas* (Döberitz-Bieszczady Mountains: p ≤ 0.001; Döberitz-Białowieska Forest: p = 0.002), *Flintibacter* (Döberitz-Bieszczady Mountains: p ≤ 0.001) and *Flavonifractor* (Döberitz-Bieszczady Mountains: p ≤ 0.001). Again, one taxon showed a contrasting tendency: *Acinetobacter* was more abundant in Döberitz than other locations (Döberitz-Bieszczady Mountains: p ≤ 0.001; Döberitz-Białowieska Forest: p ≤ 0.001) (Fig. 4).

On phylum level, the same dominant phyla occurred (Table.3). Half of the reads were assigned to Bacillota, while Pseudomonadota and Bacteroidota each made up around 17%. Actinomycetota showed a sTablele presence with low standard deviation, albeit in a lower abundance. The ratio of Bacillota to Bacteroidota was 2.51, with a relatively high standard deviation of 1.52. More varying was the abundance of families in the samples (Table.3), however, some were common in their occurrence. With more than 13%, Oscillospiraceae were the most abundant. Lachnospiraceae and Bacteroidaceae appeared at an abundance between 5 and 10%. In lower numbers but still over 2%, Prevotellaceae, Clostridiaceae, Bacillaceae and Flavobacteriaceae were present.

**Table. 3:** Relative abundance and standard deviation of bacterial taxa with a relative abundance > 2%.

Phylum	Median abundance (%)	Q1	Q3
Bacillota	43.16	39.42	51.28
Bacteroidota	20.59	14.47	26.29
Pseudomonadota	16.79	15.45	20.33
Actinomycetota	8.63	7.01	9.86
Family			
Oscillospiraceae	13.5	11.66	16.34
Lachnospiraceae	8.39	7.26	9.68
Bacteroidaceae	6.96	4.90	10.35
Prevotellaceae	3.71	2.57	4.99
Clostridiaceae	3.59	3.14	4.56
Bacillaceae	2.4	2.09	3.31
Flavobacteriaceae	2.24	1.57	2.72

### Microbiome archaea

No significant differences in the archaeal microbiome were found between the groups in alpha- or beta-diversity. Euryarchaeota strongly dominated the archaeal microbiome with over 95% median abundance. The only other phyla with an abundance over 1% were Thermoproteota and Nitrososphaerota. On family level, a dominance of Methanobacteriaceae with 75.29% of the reads was apparent. Less abundant families between 2 and 4% were Haloarculaceae, Methanosarcinaceae, Haloferaceae and Natrialbaceae. (Table.4, App.4)

**Table. 4:** Relative abundance and standard deviation of archaeal taxa with a relative abundance > 1% for phyla, respectively 2% at family level.

Phylum	Median abundance (%)	Q1	Q3
Euryarchaeota	95.56	93.30	97.49
Thermoproteota	1.93	1.24	3.11
Nitrososphaerota	1.15	0.72	1.75
Family			
Methanobacteriaceae	75.29	62.58	86.22
Haloarculaceae	3.25	1.94	4.95
Methanosarcinaceae	2.94	1.67	4.40
Haloferaceae	2.48	1.50	3.69
Natrialbaceae	2.43	1.48	3.81

### Resistome

No significant differences in resistome alpha- or beta-diversity were detected between the tested groups. With over 40% abundance each, Aminoglycoside and Macrolide, Lincosamide and Streptogramine (MLS) resistance genes were dominant in the resistome. Their associated genes equally made up a large quantity of the total, with Aminoglycoside genes (A16S: 29.20%, RRS: 6.71%, RRSH: 2.71%, RRSC: 2.64%, RRSA: 2.68%) showing a higher diversity than MLS (MLS23S: 42.21%). Tetracycline and Elfamycine resistances were two further resistance classes with a consistent abundance around 5%, each represented by only one common gene (Tetracycline – TET16S: 5.40%; Elfamycine – TUFAB: 4.54%). (Table. 5)

**Table. 5:** Relative abundance and standard deviation of resistance classes and genes. (MLS = Macrolides, Lincosamides and Streptogramines).

Class	Median abundance (%)	Q1	Q3
Aminoglycosides	44.29	43.38	45.54
MLS	42.27	40.76	43.36
Tetracyclines	5.52	5.05	5.92
Elfamycins	4.54	4.18	4.87
Gene (Class)			
MLS23S (MLS)	42.21	40.56	43.32
A16S (Aminoglycosides)	29.20	27.81	30.40
RRS (Aminoglycosides)	6.71	6.08	7.45
TET16S (Tetracyclines)	5.40	5.03	5.76
TUFAB (Elfamycins)	4.54	4.18	4.87
RRSH (Aminoglycosides)	2.71	2.63	3.01
RRSA (Aminoglycosides)	2.68	2.47	2.81
RRSC (Aminoglycosides)	2.64	2.54	2.86

## Discussion

This study investigated 78 samples of European bison and found limited differentiation of microbiome and resistome composition across locations and individuals.

### Microbiome – Bacteria

Despite the variety in sampling locations, sampling date, and individual differences, the bacterial microbiome showed limited differentiation among populations, despite considerable inter-individual variability. This pattern may be consistent with an influence of host-specific factors, including physiology and phylogeny, although additional environmental and dietary factors cannot be excluded. Indeed, gut physiology, as well as diet, have been found to be significant predictors of microbial diversity^22^. Assuming similar feeding behaviour in the animals, a limited differentiation of the bacterial composition in the gut microbiome is conceivable, subtly shaped by a varying availability of forage.

The dominance of Bacillota, followed by Bacteroidota, is a typical observation in ruminants^14,23^. Sun et al^14^. investigated the faecal microbiome of five herbivores, including European bison, in a national park in the Netherlands, which provides the only reference for this study. There, the two phyla were also dominant, however, a higher proportion of Bacteroidota was observed. The ratio of Bacillota to Bacteroidota is often used to characterize energy extraction from feed^24,25^, with a higher ratio has been associated with higher efficiency. Sun et al^14^. found a ratio of 1.3, while in this study it was 2.51 (± 1.52), showing a higher and more variable abundance of Bacillota. The most relevant distinction, though, is the use of 16S rDNA sequencing in the aforementioned study in contrast to the metagenomic approach used here. The two methods have shown to produce different proportions of taxa^26^, which is why quantitative comparisons are limited. A study on red deer, using the same methodological approach as the present one, observed a ratio of 2.44 (Lechleiter et al., under review), resembling the values reached in European bison, while roe deer only came to 1.67^17^. Roe deer are selective feeders, choosing forage which is high in sugar and protein^27^, while red deer as mixed feeders^28^ and European bison, as browsers^29^, ingest a high proportion of fibre. These dietary differences may contribute to the observed variation in the Bacillota-to-Bacteroidota ratio. Pseudomonadota are found in the microbiome of European bison and cattle^14,30^. They have been found in neonatal dairy calves^31^, suggesting a ubiquitous occurrence, but increased numbers also seem to be connected to inflammatory response^32^ and pathogen presence^33^. While single samples had a slightly increased ratio of Pseudomonadota in other locations, most apparent differences were visible in Döberitz, a finding which will be discussed later. The fourth phylum present in higher abundance were Actinomycetota, which have been found in roe deer before^17^ and contain species linked to cellulose and lignin degradation^34^.

At the family level, the bacterial microbiome composition was more distinct. Oscillospiraceae were dominant, as has been described in European and American bison before^14,35^. They have been associated with cellulose digestion^36^ and are therefore an important part of the ruminant microbiome. Several more of the common families contain taxa with similar properties, like some Lachnospiraceae, providing cellulose and fibre fermentation^36^ and Bacteroidaceae, digesting polysaccharides^36^. Other families were less abundant but still commonly present at over 2%. Prevotellaceae, which show ubiquitous occurrence all throughout the bovine gut^37^ have also been found in the rumen microbiome of the American bison^35^. The genus *Prevotella* in this family has been connected to protein digestion^38^. Clostridiaceae, a family with members colonizing the gastrointestinal tract of animals^23^, has been found to be inversely connected to starch ingestion in herbivores^39^. An interesting finding is the family of Bacillaceae. This family is not usually mentioned as part of gastrointestinal microbiomes but does appear in small abundance in cows^40^. Due to its capability to synthesize digestive enzymes and gut barrier enhancement, the genus *Bacillus*, which has been found in European bison and cows by Sun et al^14^., is being supplemented in cattle to improve health and performance^41^. Overall, this composition agrees with the phylogeny and diet of the European bison.

Sex and age were not found to have significant influences on the microbiome and resistome composition. One reason for this lack of effect may be, that highly abundant genes cover up variations in rarer ones. While information on the European bison is sparse, in another family of wild ruminants, the cervidae, the data is varied. While sex had an influence on the microbiome composition in free-living deer, a study on captive white-tailed deer did not find differences between male and female individuals^33^. In roe deer, cefpodoxime resistant *E. coli* were significantly more abundant with increased age^42^. This pattern, however, did not appear in other deer species or resistance types. Generally, exposure is considered a determining factor for resistance gene abundance^34^.

The highest number of compositional differences was found in the bacterial composition of the Döberitz samples. The most probable source of this phenomenon is that these samples were the only ones which were collected from droppings, as opposed to samples collected directly from animals. Changes in the proliferation rate of the taxa due to vastly different environmental conditions and colonization by environmental organisms are to be expected and might be reflected in the significantly higher richness. Interestingly, these differences were not visible on phylum level, where neither the relative abundance of taxa nor the ratio of Bacillota to Bacteroidota showed a significant change. While Pseudomonadota were present in a higher abundance, this trend was not statistically significant. On family and genus level, though, differences between locations became evident. Several anaerobic colonizers of the gastrointestinal tract were significantly lower in abundance in Döberitz, in comparison to other locations. Moraxellaceae, on the other hand, a family within the Pseudomonadota, mostly represented by the genus *Acinetobacter*, appeared in significantly higher abundance in Döberitz. This aerobic genus is an inhabitant of animal microbiomes but also soil and other environmental habitats^43^. These variations to the other locations suggest a shift of the microbial community towards aerobic taxa after defecation, lowering the comparability of samples collected from the environment in comparison to those which are taken directly from animals. Apart from Döberitz, the other locations differed slightly in the abundance of the most common taxa, likely because of individual variations, but no significant difference appeared.

### Microbiome – Archaea

As gastrointestinal microbiomes tend to have a dominance of bacteria over other organisms^14^, the distinctly lower number of archaeal reads in this study was to be expected. This low abundance may be a reason why no significant differences were found between groups. Moreover, all samples were taken from the same species and host phylogeny was found to have a higher influence on the archaeal microbiome composition than diet^44^. Euryarchaeota dominated the dataset with over 95% abundance, making for a low diversity of taxa, a result similar to the findings of Peng et al^45^. in cattle. This phylum has also been found in faecal samples of cows^46^ and is mostly represented by the family of Methanobacteriaceae in the present study. Methanobacteriaceae have also been reported in the American bison^35^ and pasture grazing cattle^47^ and, together with other methanogenic taxa, they form a large part of the herbivore microbiome^48^. With an abundance in animals with a high fibre diet^48^, they possibly aid polysaccharide degradation by reducing hydrogen^45^. Since most studies are based on 16S rDNA for taxonomic investigation, the taxonomic resolution will be reduced in comparison to a metagenomic approach^49^. In the present study, rarer archaeal taxa could be found in the dataset, that provide deeper insights into the microbial composition. NoTablely, some halophilic families like Haloarculaceae, Haloferaceae and Natrialbaceae were present. The presence of halophilic archaea in animal microbiomes is commonly reported^50^ but the biological reason for their presence is yet unclear. A study on roe deer in Germany hypothesizes a connection to salt ingestion from mineral licks^17^, which are also provided for the European bison herd in Poland, but the consistent finding of these organisms in wild animals poses an intriguing phenomenon. The knowledge about archaea in wildlife is limited and the use of methods like metagenomic sequencing offers insights that may one day be valuable in forming a bigger picture.

### Resistome

With no significant differences between groups, the European bison resistome was dominated by genes for Aminoglycoside and MLS resistances. Aminoglycosides are commonly used in veterinary medicine^51^ and resistances to Streptomycin, an antibiotic within this class, have been found in Polish wildlife^52,53^. A cultivation of *Escherichia (E.)* *coli* from faecal samples of European bison from Lithuania revealed gentamicin resistance in 50% of the resistant isolates^54^. Resistance genes against agents in the class of MLS, common in the treatment of livestock^51^, appear in wild boar in Poland^53^. Both classes are commonly found in European wildlife^17,55^. Other investigations of antimicrobial resistances in European bison microbiomes were performed by isolating *E. coli* from faecal samples and testing the isolates for specific resistances^16,56^. While three samples from sanitary culling in Białowieska Forest did not yield any non-wildtype isolates^16^, the study in Lithuania found at least one resistance in 44% of the cultivated *E. coli*^56^, demonstrating the heterogeneity of such investigations based on methodology and biological factors. The cultivation method can offer a specific insight into functional resistances, metagenomic sequencing, on the other hand, allows for an overview of the genetic potential in a microbiome. Tetracycline resistance genes were found in lower abundance. This antibiotic class is commonly used, especially in cattle^51^, and can persist in soil, inducing the rise of resistance genes^57^. Contact with soil-associated microbiomes, for example through grazing, may expose animals to resistance genes. Overall, the resistome composition was very similar to that described in red deer from Germany (Lechleiter et al., under review), suggesting that wild ruminants in Central Europe may harbour comparable resistome profiles. The detected resistance genes may reflect both naturally occurring components of the gastrointestinal microbiota and potential environmental influences. While metagenomics offers diverse insights into the taxonomic and genetic composition of a population, highly abundant genes may overshadow rare ones. This is another potential reason for the occurrence of mostly the same dominant bacterial families. While the presence of AMR genes cannot be equated to active resistance, it can serve as an indicator of the genetic potential of the microbial community. The occurrence of these genes in several populations of European bison with different management styles suggests a ubiquitous distribution through shared environmental exposure pathways or sTablele background reservoirs of antimicrobial resistance genes.

Despite substantial inter-individual variability, both the microbiome and resistome exhibited limited differentiation among the investigated populations. This suggests that factors shared in European bison populations, including host physiology, phylogenetic relatedness, and broadly similar feeding ecology, may contribute to the observed microbial composition. Host-associated factors have repeatedly been identified as important drivers of microbial diversity^10,22^, while diet is known to shape ruminant microbiomes^13^. European bison populations occupy comparable ecological niches^29^ and may therefore experience similar dietary conditions, including supplementary feeding in managed populations. Dietary composition has been shown to influence ruminant microbiomes, while host-associated factors are recognized as important determinants of microbial diversity^14,58^.

## Limitations

Due to the sampling of droppings in Döberitz, multiple sampling of the same individual cannot be excluded. This likely skewed the dataset from that location. A seasonality of the microbiome composition has been described in wild ruminants^58^, making it plausible that some of the variation within the dataset originated in differences of sampling time. As only the sampling year is known, this variable could not be accounted for. Although substantial variability was observed among individual animals, no clear population-specific clustering was detected. As microbiome relative-abundance data are compositional in nature, the applied statistical analyses should be interpreted as exploratory and descriptive. Taxonomic profiling was performed using Kraken2. While methods such as Bracken may improve abundance estimates by redistributing ambiguously assigned reads, the present analyses focused on broad taxonomic patterns and dominant taxa, for which the influence on the main conclusions is expected to be limited. Sampling under field conditions can lead to different time frames until the sample can be properly stored. Carrol et al^33^. found that the microbial composition in a stool sample did not change significantly within 24 hours of storage at room temperature, a time frame which has not been exceeded in the present study. The observed microbial composition is, to a certain degree, always influenced by the method of DNA extraction^59^ and annotation^60^. When interpreting and comparing results, this needs to be regarded.

## Conclusions

Despite substantial inter-individual variability, the investigated European bison populations exhibited limited population-level differentiation and shared dominant microbial and resistome taxa. Unlike previous 16S rDNA studies, shotgun metagenomics enabled simultaneous characterization of bacterial, archaeal, and resistance gene profiles. Future, species-level analyses, may further expand on these findings. The bacterial microbiome composition is comparable to that of other ruminants, including taxa involved in cellulose digestion and polysaccharide degradation. Differences in the ratio of the dominant phyla, Bacillota and Bacteroidota, may be related to the changing fibre availability throughout seasons and habitats. Most prominent were the compositional differences of the Döberitz samples, where aerobic taxa dominated. As samples at this location were the only ones taken from droppings, changes in the microbiome may be induced by environmental influences and contamination. This investigation produced novel insights into the bacterial and archaeal microbiome as well as the resistome of the European bison.

## Supplementary Information

Supplementary Information 1.

Supplementary Information 2.

Supplementary Information 3.

Supplementary Information 4.

Supplementary Information 5.

## Data Availability

The datasets generated and analysed within this study are available in the European Nucleotide Archive repository with the primary accession code PRJEB108958.
