# Supplementary material for: Microbiome and resistome of the European bison (Bison bonasus)
Source: Sci Rep. 2026 Aug 6;16:24310. doi: 10.1038/s41598-026-64319-9 (PMC13448691; doi:10.1038/s41598-026-64319-9)
Supplement: Supplementary file 1 [file 41598_2026_64319_MOESM5_ESM.docx]

Appendix

App.1: Metadata

App.2: Sequencing depth per sample

App.3: Non-significant results of beta-diversity analyses. (F = 3.06, R^2^ = 0.177, p = 0.007)

App.4: Relative abundance of archaeal families per location. Bubble size corresponds to abundance. Families with an abundance of <2% per sample are summarized in “<2”.
